# Replacing Animal Testing with Stem Cell-Organoids : Advantages and Limitations

**DOI:** 10.1007/s12015-024-10723-5

**Published:** 2024-04-19

**Authors:** Guiyoung Park, Yeri Alice Rim, Yeowon Sohn, Yoojun Nam, Ji Hyeon Ju

**Affiliations:** 1 School of Biopharmaceutical and Medical Sciences, Health & Wellness College, Sungshin Women’s University, 55, Dobong-ro 76ga-gil, Gangbuk-gu, Seoul, Republic of Korea; 2 CiSTEM laboratory, Convergent Research Consortium for Immunologic Disease, Seoul St. Mary’s Hospital, College of Medicine, The Catholic University of Korea, Seoul, 06591 Republic of Korea; 3 Division of Rheumatology, Department of Internal Medicine, Seoul St. Mary’s Hospital, Institute of Medical Science, College of Medicine, The Catholic University of Korea, 4 3, Seoul, 06591 Republic of Korea; 4 Department of Biomedicine & Health Sciences, Seoul St. Mary’s Hospital, College of Medicine, The Catholic University of Korea, Seoul, 06591 Republic of Korea; 5 Department of Biohealth Regulatory Science, Sungkyunkwan University, Suwon, South Korea; 6 Yipscell Inc, L2 Omnibus Park, Banpo-dearo 222, Seocho-gu, Seoul, Korea

**Keywords:** Animal Testing Alternatives, Animal Testing law, Stem Cell, Organoids, Organ-on-chips, iPSC

## Abstract

Various groups including animal protection organizations, medical organizations, research centers, and even federal agencies such as the U.S. Food and Drug Administration, are working to minimize animal use in scientific experiments. This movement primarily stems from animal welfare and ethical concerns. However, recent advances in technology and new studies in medicine have contributed to an increase in animal experiments throughout the years. With the rapid increase in animal testing, concerns arise including ethical issues, high cost, complex procedures, and potential inaccuracies.

Alternative solutions have recently been investigated to address the problems of animal testing. Some of these technologies are related to stem cell technologies, such as organ-on-a-chip, organoids, and induced pluripotent stem cell models. The aim of the review is to focus on stem cell related methodologies, such as organoids, that can serve as an alternative to animal testing and discuss its advantages and limitations, alongside regulatory considerations.

Although stem cell related methodologies has shortcomings, it has potential to replace animal testing. Achieving this requires further research on stem cells, with potential societal and technological benefits.

**Graphical Abstract:**

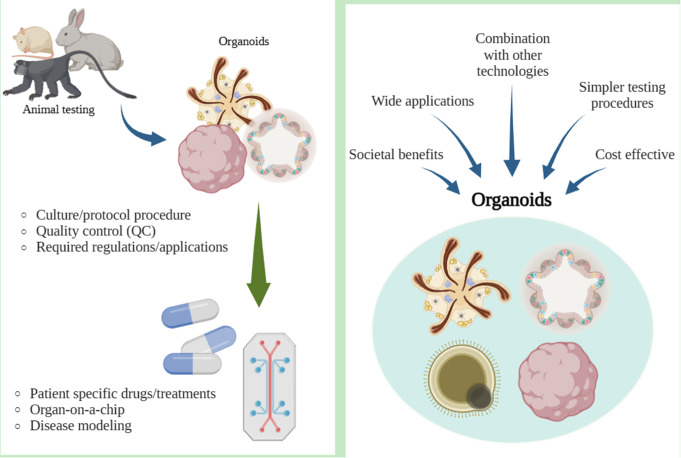

## Introduction

Historically, animal models have contributed substantially to the advancement and study of vaccines, surgical techniques, and various scientific experiments [1]. However, owing to the problems associated with animal testing, researchers are now questioning whether animal models and tests are the best options for these procedures. Growing animal testing is ethically concerning amid scientific evolution. According to the Humane Society International Organization, more than 100 million animals are killed annually worldwide for scientific purposes (Humane Society International). The animals used vary depending on their traits and include rats, mice, rabbits, dogs, cats, guinea pigs, zebrafish, swine [2, 3].

In December 2022, the U.S. Food and Drug Administration (FDA) announced animal testing is no longer mandatory safety approval of products [4]. However, products that are used on the human body still require safety testing. In other words, testing for toxicity, compatibility, and safety is compulsory for products; however, animal testing is unnecessary for conducting these tests. In response, research facilities and companies have introduced alternatives such as computer simulations and *in silico* models. Stem cell therapy has gained popularity throughout the medical field, and various studies are underway to gain deeper knowledge [5]. With the emergence of this stem cell-based test, alternative methods have also arisen, potentially offering to become a replacement for animal testing.

When comparing test options, alternatives offer more beneficial attributes than animal testing. Non-animal tests are cost-effective, less time-consuming, and simpler procedures than animal tests [6]. However, most research institutions use animal models. This is because animal testing has been a longstanding experimental approach for decades [7, 8]. Efforts are being made to replace animal testing with the use of human cells, as animal testing results often exhibit interspecies differences with humans, thus lacking the ability to reliably predict clinical outcomes. Application of advancing stem cell technology continue, but completely replacing animal experimentation poses significant challenges. Therefore, it is important to conduct further studies to advance the science of alternative testing methods. This review aimed to summarize the use of stem cell technology as an alternative to animal testing and discuss its advantages and limitations.

## Current State of Animal Testing

### Uses of Animal Testing

Animal testing has been used for decades, and in the 21st century, the number of tests has increased considerably [2]. With approximately 100 million animals used for testing annually worldwide, science has been rapidly evolving. The primary function of animal testing is to test drugs, their toxicity, and their compatibility with the human body to ensure safe use. Hence, pre-launch testing is crucial. Companies and research facilities must subject their products to clinical trials before introducing them to potential customers.

Neurological disorder such as Parkinson’s and Alzheimer’s have also been modeled in animals to understand their mechanisms and to determine suitable treatments [9–11]. For instance, in the case of Parkinson’s disease, various animal models have been employed, including Caenorhabditis elegans, Zebrafish, and mice. Additionally, genetically modified mice carrying mutations associated with proteins like α-synuclein, Parkin, Pink1, and LRRK2, as well as mice induced with α-Synuclein Pre-Formed Fibril (PFF), are utilized to assess dopaminergic neuronal loss and investigate changes in α-synuclein aggregation. In Alzheimer’s disease, transgenic mice carrying mutations associated with familial Alzheimer’s disease (FAD), such as the 5xFAD model, are commonly used. These models allow for the evaluation of amyloid beta reduction through histological methods and the assessment of drug efficacy using behavioral tests like the Maze, providing insights into underlying disease mechanisms. Animals utilized as disease models contribute significantly to our comprehensive understanding of the mechanisms behind various illnesses, facilitating our grasp of these conditions. Research conducted using these animal disease models has indeed contributed to the discovery and development of treatments. However, it’s scientifically crucial to acknowledge that these animal models often present disparities in lifespans compared to humans and may not entirely mirror the intricate etiology of human diseases. Additionally, while animal experimentation is utilized for various conditions such as cancer, diabetes mellitus, and traumatic brain injury, it’s constrained by its inability to fully capture the nuances of the human immune system and intricate disease mechanisms (Table 1).

**Table 1:** Current animal models for diseases/conditions including their limitations

Disease	Animal model currently in use	Limitations	References
Parkinson’s Disease	Non-human primates *Caenorhanditis elegans* *Drosophila melanogaster* Zebrafish Rodents (mice, rats)	Time consuming Complex procedure Different from humans Lacking Synuclein homolog Gene research still in progress Expensive	[10, 11]
Alzheimer’s disease	Rodents (mice, rats)	Cannot completely mimic patient pathophysiology(no complete cure yet)	[9]
Cancer	Rodents (mice, rats) Zebrafish Fruit flies	Small size animals (limited blood supply) Difference in physiology, immunity, heredity from human	[12]
Diabetes mellitus	Rodents (mice, rats, hamsters) Pigs	Difference in concentration of blood glucose levels from humans Complex disease mechanism and procedure	[13, 14]
Traumatic brain injury	Rodents (mice, rats)	Different complexity and size compared to human brain Gene expression varies from that of humans	[15]
Wound healing	Rabbits Rodents (mice, rats) Pigs	Anatomical and physiological difference from that of humans Lacks design and procedures related to standardization	[16]
Skin/eye irritation	Rodents (mice, rats) Rabbits	Chemical misclassification possibility (owing to difference with humans)	[17–19]

In addition to modeling diseases, animals are also used to test cosmetics or healing rates of products. In the cosmetics industry, animals are typically used to test skin or eye irritation to assess the safety of these products in humans [17, 18]. The Draize test, developed in 1944 to test for such hazards in rabbits [19], is used to test products such as drugs and balms for wound healing. It involves creating wounds on animals to gauge recovery rates [16].

### Related laws, Guidelines, and Principles

As of 2023, current regulations state that the FDA no longer deems animal tests necessary for evaluating product safety [4]. This enables companies and research facilities to explore possible non-animal testing when obtaining product approval. Additionally, out of 195 countries worldwide, only 42 have laws or regulations limiting animal testing for products (The Humane Society). Animal testing laws have been implemented by banning animal testing or limiting its use during testing. Europe completely banned cosmetics tested on animal testing in 2013 [3, 20, 21]. This demonstrates a push to limit animal testing; however, the movement remains ineffective because of the absence of laws against animal testing in most countries.

**Fig. 1:**
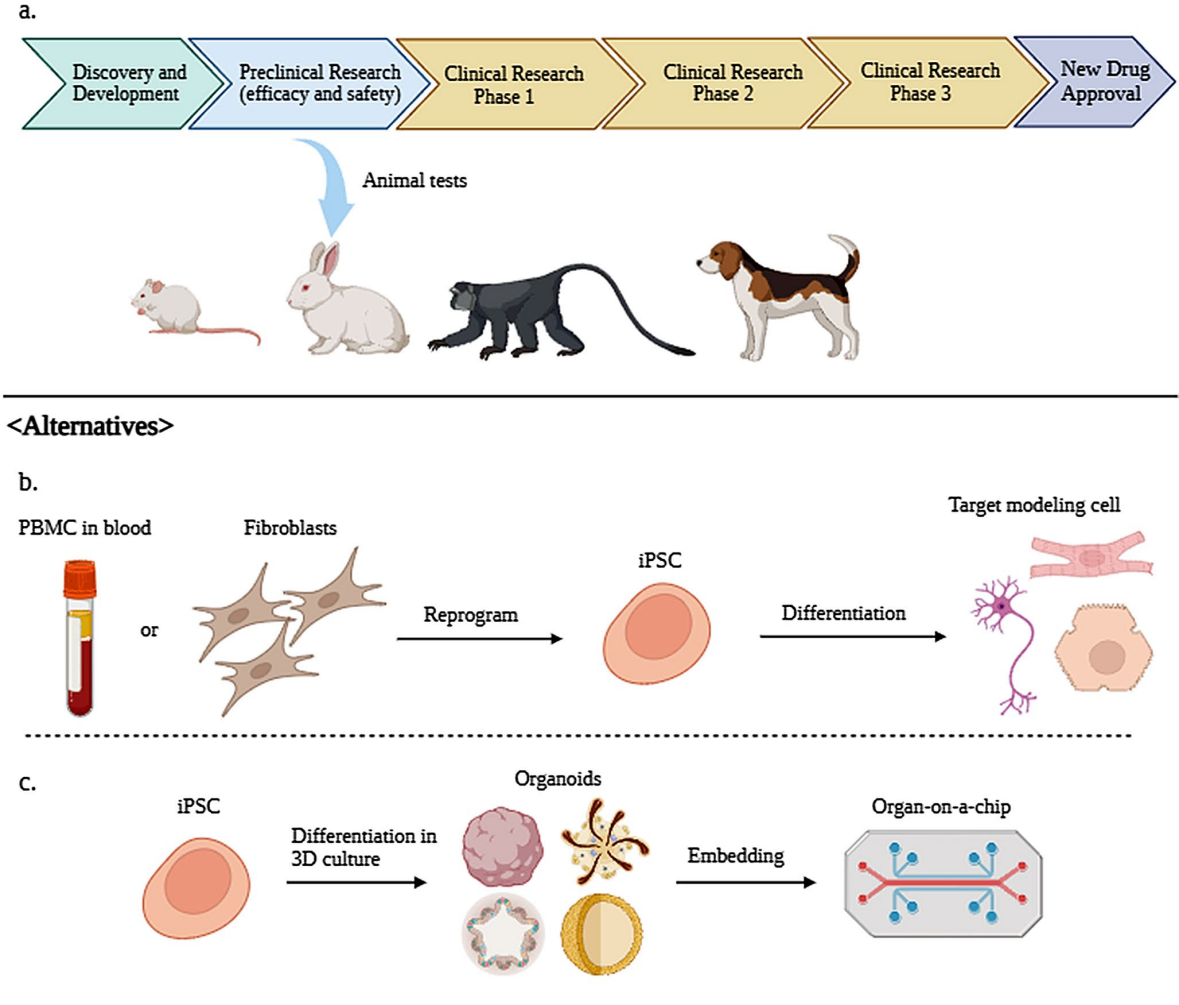
(**A**) Procedure of new drug approval as stated by the Food and Drug Administration (FDA). In the preclinical research stage, small, medium, and large animals are usually used for testing new drugs. (**B**) iPSCs that can replacing animal testing. PBMCs or fibroblasts are reprogrammed to iPSCs and subsequently differentiated into target modeling cells such as neurons, cardiomyocytes, and hepatocytes. (**C**) iPSC-derived 3D organoids enable in vitro efficacy and safety testing. Organ-on-a-chip embedded with organoids used in in vitro tests, created using BioRender

Guidelines for animal experimentation and clinical trials for drug development and safety testing have varied procedures among companies and researchers up to now. So, the Guidance for Industry for Preclinical Safety Evaluation of Biotechnology-Derived Pharmaceuticals from the Center for Drug Evaluation and Research provides guidelines for the safety assessment of products compiled from regulatory standards of several countries. According to these guidelines, preclinical trial researchers should consider factors such as animal species, age, delivery method (dosage, administration, treatment regimen, etc.), and test material stability [22] (Fig. 1).

The FDA has also provided a drug development process that includes these steps. The first step in drug development is discovering and researching a new drug (discovery and development stage). The second stage is preclinical research, in which drugs have to undergo a series of animal tests (or alternative tests, if possible) for safety. The FDA strongly suggests that animal preclinical trials follow Good Laboratory Practice (GLP). The main elements of GLP are as follows [23]: appropriate use of qualified personnel, quality assurance, appropriate use of facility and care for animals, proper operating procedures for animals used in trial, individual animal data collection and evaluation, testing product properly handled and analyzed, study proceeds with an approved protocol, data should be collected as outlined in the protocol, and full report prepared after procedures.

To enhance clinical translation, reproducibility issues in preclinical trials, such as biased allocation, insufficient controls, and lack of interdisciplinary, uncharacterized, or poorly characterized supplies [24]. The third step involves clinical testing on humans to assess safety and efficacy. The fourth and fifth stages comprise FDA post-market safety monitoring for all approved drugs [25].

Guidelines also suggest the 3R (replacement, reduction, and refinement) principle, which recommends that scientists follow certain criteria during clinical trials. Replacement involves using other testing methods other than animal testing [26]. In computer models, tissues, or stem cell research, if alternatives to animal testing exist, researchers should prioritize their use. Reduction involves minimizing the number of animal tests [26]. Questioning the necessity of animal tests during a particular part of our research and reducing their numbers imbues the concept with meaning. Refinement focuses on minimizing stress and providing the best care to animals [26], including providing proper food, entertainment, and clean well-maintained shelters.

As International efforts for animal replacement methods, research and development into alternative testing methods is already underway in both Europe and the United States, with each regulatory body establishing its own initiatives. In Europe, the European Center for the Validation of Alternative Methods (ECVAM) was founded in 1992, and since 2013, the sale of cosmetics containing ingredients tested on animals has been completely banned. Moreover, there are plans to expand the scope to include medical devices, health supplements, and pharmaceuticals in the future. In the United States, the Interagency Coordinating Committee on the Validation of Alternative Methods (ICCVAM) was established in 2000. The objective is to reduce animal testing by 2025 and eliminate mammalian animal testing entirely by 2035 through innovative advancements in alternative testing methodologies. In 2022, amendments to the Food, Drug, and Cosmetic Act in the United States removed mandatory animal testing requirements in the drug development stage and presented alternative testing methods as viable non-clinical trial options.

### Problems/limitations of Animal Testing

A pressing issue with animal testing is the ethical concerns stemming from it. Most studies have demonstrated that these models undergo invasive procedures that often result in pain or even death. Research indicates that animals share pain and emotional capacity with humans [27]. Thus, sacrificing them for research can appear cruel. Advocates call for equitable treatment, opposing animal testing as inhumane and cruel. Such ethical issues has always followed animal testing and are ongoing [28].

Moreover, some studies have indicated that animal testing is not an accurate model for medicines or substances, highlighting the need for accurate and efficient testing alternatives that are similar humans. The complexity of human disease mechanisms raises doubts whether animal models can accurately replicate them.

Physiological differences between animals and humans mean a product safe for animals may not guarantee human safety [29]. Interspecies differences have led to poor results in correlating animal testing with human outcomes, consequently causing several clinical trial failures [30]. Between 2010 and 2017, clinical trials for drugs had a greater chance of failing phase І, owing to safety and efficacy [31]. In addition, even if a product passes phase І there is still a 90% rate of failure while undergoing the necessary procedures [32, 33]. Prolonged use of animal testing can ultimately endanger humans, as some drugs and products approved through trials were later deemed harmful. Concerns such as high cost and long laborious procedures will be discussed below.

## Benefits of Replacing Animal Testing

The main benefits of replacing animal tests with alternatives are as follows: cost-effective, time efficient, less complex testing procedures, and societal benefits.

Stem cell modeling is less expensive than animal testing. The Draize test mentioned before costs approximately $1,800, whereas non-animal testing methods cost considerably less [6]. Affordable procedures offer renewed chances for past costly research to emerge. A decrease in the cost of procedures would facilitate new drug development, making opportunities for new technologies easier.

Animal testing requires prior preparation that is often complex and time consuming. Several guidelines of various organizations worldwide follow certain principles and procedures. For animal testing, factors such as providing clean and well-maintained shelters, food, necessary supplies for survival, and entertainment are laborious [26]. Alternatives are time-efficient and less laborious, simpler protocols, and fewer supplies to maintain procedures.

## Alternatives to Animal Testing Related to Stem Cells

### Organoids

Organoids are organ-like structures derived from self-organizing stem cells in 3D cell cultures. They exhibit organ-specific characteristics and originate from stem cells undergoing self-organization [34, 35]. . They are beneficial over previous 2D cell culture, as they can show near-physiological cellular composition and actions [36]. Organoids are typically established from embryonic stem cells (ESCs), human pluripotent stem cells (PSCs), and adult stem cells [37–39]. The potential of organoids as alternatives stems from their correlation with patient reactions to products such as drugs, indicating that they are a promising for rare diseases where clinical trials are impractical [39]. Organoids have a wide range of applications and are suitable for studies of infectious diseases, hereditary diseases, and toxicity, and can provide personalized medicine for individual patients [38].

Recent studies have shown that PSC organoids can form complex brain organoids that are useful for modeling traumatic brain injury [15]. Organoids derived from PSCs are of various types, including stomach, lung, liver, kidney, cerebral, and thyroid, and can contribute to organ failure or dysfunction. Cancer organoids are cultured from thin tumor sections, which are efficient for studying cancer syndromes [34]. Organoid studies on Alzheimer’s disease highlight the possibility of using familial or sporadic Alzheimer’s disease induced pluripotent stem cells (iPSCs) to model brain activity [40]. Thyroid follicles derived from hESCs have the potential to be used as organoids to treat hypothyroidism [41] (Table 2). Technology development of 3D bioprinting organoids is underway, promising better productivity. Bioprinting for organoids includes inkjet-based bioprinting, laser-assisted bioprinting, extrusion-based bioprinting, and photo-curing bioprinting [42]. Ongoing studies are also exploring 3D printing technology using organoids, offering the possibility of creating organs for patient-tailored services and toxicology research.

**Table 2:** Methods and considerations of QC for organoids

Methods	Detailed examples of methods	Functions	References
Imaging technology	Bright-field microscopy Live cell imaging Transmission and scanning electron microscopy Immunofluorescent imaging	Assess morphology Size examination Quantity assessment Determine specific functions	[65, 66, 69]
Gene expression analysis	Single or bulk RNA sequencing Quantitative PCR(qPCR)	Evaluate expression of genes Reveal cell identity and composition	[65]
Assay methods	ELISA assay	Secretome quantification	[65]
	Luciferase assay	Measure enzyme activity	[65, 68]
Staining	Glycosaminoglycan(GAG) staining	Show presence and distribution of glycosaminoglycans within extracellular matrix of organoids Provide insight to composition and function of the organoid microenvironment	[68]
	Immunostaining	Identify and localize proteins of interest Allow to study protein interactions in organoids	[65, 68]
	Alizarin red staining	Visualize calcium deposits(mainly used in bone tissue engineering in organoids)	[69]
Implant method	Mouse transplantation	Assessment of in vivo functions	[65, 68, 70]
Extracellular microenvironments	Soluble bioactive molecules Extracellular matrix Biofluid flow	Growth rate Contributes to formation of organoids	[71]

However, organoids still possess limitations that render them unsuitable tools to replace animal testing. Organoids lack of vasculature structure affects growth and maturation, leading to differences in behavior compared to the original tissue [59]. This may result in only partial replication, leading to an incomplete disease model [38]. Moreover, the complexity and heterogeneity of certain organs, such as the brain or immune system, pose challenges for complete replication in organoid models. This inability to replicate such complexity can affect the translatability of findings from organoid studies to clinical applications. Research and experiments involving organoids often require lengthy culture protocols, which can vary depending on the type of organoid being cultivated. In some extreme cases, organoid culture may extend for months or even years, as seen in examples such as intestinal organoids(8 weeks or more), retinal organoids(6 ~ 39 weeks or more), brain organoids(12 weeks or more), and liver organoids(4 ~ 8 weeks or more) [60–64]. Even after going through the lengthy process, there are sometimes a lack of established organoids in sufficient numbers. This limited availability of organoids can hinder the procedure of functional testing, which can lead to insufficient research outcomes. Organoids also lack the intricate network of connections that can be seen in living organisms. Inter-organ communication is crucial when checking metabolic health, and with organoids lacking such an important factor, it is difficult to create treatments for any abnormalities regarding infection and diseases. Organoids also lack a diverse set of cell types, structural organization, and physiological functions in comparison to functioning organs, which limits the ability to accurately replicate disease processes and responses to treatment [59]. When compared to animal models, organoids fall behind, as animal models offer a broader view of processes for diseases, immune responses, and systemic effects of treatments. Another noteworthy concern arises from the fact that current production technology for organoids under GMP (Good Manufacturing Practice) standards has yet to be established.

#### Quality Control of Organoid

For organoids to serve as suitable models for diseases or experimental purposes, quality control (QC) is essential. Accuracy and consistency in production lead to more precise results, ensuring better therapeutic treatments or modeling. If quality control for organoids isn’t established sufficiently, problems such as inconsistent test results, misinterpretation of existing data, wastage of valuable resources, reproducibility issues, unreliable models, and ethical concerns regarding biomedical studies could arise.

Organoid structures and functions can be assessed through multiple methods. Structural assessment of organoids can be performed using bright-field imaging for both quantitative and qualitative research. Additionally, methods such as immunofluorescent staining, transmission electron microscopy, and scanning electron microscopy are also utilized [65, 66]. The functionality of organoids can be assessed through qPCR and single-cell or bulk cell RNA sequencing, which provide quantitation of marker gene expression, revealing cell identity and composition [67]. Assay methods like ELISA and colorimetric assays are useful for secretome quantification while Luciferase essays help measure enzyme activity [65, 68]. Staining methods such as Glycosaminoglycan (GAG) staining(specifically for synovial mesenchymal stromal cell (SMSC) organoids), immunofluorescence staining, and Alizarin red staining mainly help with visualizing components within the organoid [65, 68, 69]. There are also more direct methods like implantation to test the in vivo functions of organoids [65, 70] (Table 3).

**Table 3:** Possible alternative tests/models to replace animal testing and cure diseases

Disease	Stem cell related tech	Cell types/Modeling	Advantages	Limitations	Reference
Parkinson’s disease	iPSC-derived model	Dopaminergic neurons (DAn)of the substantia nigra pars compacta (SNpc)	Patient cell driven Compatible for large screening Cost efficient	Lacks complete physiological network connections that imitate brain	[11, 43–45]
Cardiac disease	iPSC cardiac myocytes	Cardiac progenitors (human HCN4+) ESC derived ROR2+, CD13+, KDR+, PDGFRα+	Useful when analyzing mechanisms underlying disease Suitable for combined image-based deep learning or machine learning data analyses	Possible graft-related ventricular arrhythmia Limited engraftment of injected cardiomyocytes	[46–48]
Cancer	Cancer organoids	Culture of thin tumor sections	Effective to study human cancer syndromes Aides in overcoming traditional cancer cell line systems	Needs successful engraftment, Technical challenges Variable growth rates	[34, 49–51]
	Cancer-on-a-chip	Endothelial cell lined gel embedded with bone stem cells (attracts cancer cell extravasation)			
	iPSC models	Reprogrammed tumor specimen iPSCs with premalignant or early genetic lesions			
Alzheimer’s disease	iPSC-derived brain cells	Neurons Astrocytes Microglia Oligodendrocytes Pericytes Vascular endothelial cells	Able to replicate specific traits of human brains Brain modeled with functional blood-brain barrier	Bettering the consistency and reproducibility still in task Variability of clones reported from same parental iPSC line	[9, 40, 52]
	Organoids	Familial Alzheimer’s disease iPSCs Sporadic Alzheimer’s disease iPSCs			
Organ failure/dysfunctions	Organ-on-chip	Lung-on-a-chip Intestine-on-a-chip Kidney-on-a-chip Heart-on-a-chip	Promising organ source when in shortage Organ rejection risk lowered Provides safe drug screening	Devising engraft cell lines required Interpreting genetic, epigenetic, and clone variation remains inexperienced after results.	[50, 53]
	Human iPSC	Endogenous or exogenous stem cells (lung regeneration) Liver hepatoblasts (hepatotoxicity)			
Traumatic brain injury	Human cerebral organoids	iPSCs originated from human dermal fibroblasts	Able to replicate specific traits of human brains	Does not possess all brain cell types Lacks vasculature	[15, 54]
	Brain-on-a-chip	3D cultured iPSC-derived neural progenitor cells			
Hypothyroidism	Thyroid organoids	Human thyroid follicles from hESCs	Possible signs of effective therapy Models provide insight into thyroid development	Limited efficiency after input Requires extended time to show results Limited blood vessel presence shown	[41]
Skin/eye irritation (or injury)	Organ-on-a-chip	Eye-on-a-chip, Skin-on-a-chip	Able to replicate movement (eye blinking) or reaction with chemicals	Limitations when verifying differentiation	[55, 56], [57, 58]
	Skin organoids	KRT5+, KRT15+, CD49F + epidermal KRT15 + peridermal PDGFRα+, P75 + Dermis			

Extracellular microenvironment, which contain such things as soluble bioactive molecules, extracellular matrix, and biofluid flow, contributes to the growth rate and formation of organoids. Given the variation in extracellular microenvironments across different types of organoids, it is imperative to modulate the extracellular microenvironment accordingly for each organoid type. This ensures the production of organoids with consistent quality across different production batches [71].

#### Regulations/Applications Regarding Organoids from the FDA

While there aren’t any specific regulations regarding organoids from the FDA(Food and Drug Administrations) as of in the recent years, there are two categories of applications that include framework for cell related therapies, which include organoids. There are two applications, Biologics License Application (BLA) and the Investigational New Drug (IND) Application. The BLA, as stated in the official website of FDA, is a request for permission to introduce and deliver for a biologic product(vaccines, somatic cells, gene therapy, tissues, recombinant therapeutic proteins, organoids, etc.) into interstate commerce. Requirements for a BLA includes applicant information, product/manufacturing information, pre-clinical studies, clinical studies, and labeling. The IND application is a request for authorization to administer an investigation drug or biological product to humans. IND had three types: Investigator IND, Emergency Use IND, and Treatment IND which could fall into two categories being commercial or non-commercial. The IND application must contain the following broad areas of information: Animal Pharmacology and Toxicology studies, Manufacturing Information, Clinical protocols and Investigator Information.

When examining the current ongoing clinical trials(*ClinicalTrials.gov*) in the application of organoids, it can be noted that they are being utilized in refractory cancers, osteosarcoma, high-grade glioma, advanced breast cancer, and colorectal cancer. This pertains to the utilization of the organoid platform to investigate the sensitivity to various drugs (chemotherapy, hormonal therapy, targeted therapy) by exposing them to each individual agent (or combination of agents). It is anticipated and ongoing to aid in clinical decisions regarding the optimal treatment option for each patient.

### Organ-on-a-chip

Organoid chips(OoC) can be regarded as the outcome of merging biology and microtechnology, serving as microfluidic cell culture devices [72, 73]. OoC has the ability to mimic the cellular environment, which leads to an examination of their effects on cell communication with more accessibility and ease. The chips are generally designed by collecting cells (primary cells, transformed cell lines, human ESC, or iPSCs) using equipment with pumps(that enable fluid flow), incubators, sensors, and microscopes to monitor and examine the cells in the system [49, 74] (Fig. 1). Depending on the type or cell or method cells can be aggregated in matrix or matrixless conditions [75].

Various types of human organ chips, including the liver, heart, eyes, kidneys, bones, intestines, and skin, are used to simulate the breathing motion. Single-organ chips such as liver-on-a-chip and lung-on-a-chip are useful for observing individual chemical reactions [53]. There are also multiple organ-on-chip, which are organ-chips connected to a vast system [76]. The main purpose of multi-organ-on-chips is to simulate the entire body, recognizing that a single organ does not represent the entire human system. Using multiple organ-on-chips connected to one system allows the analysis of how various organs communicate with each other.

The U.S. Food and Drug Administration (FDA) and the U.S. National Institutes of Health (NIH) have provided project support for tissue chips for drug screening, including lung-on-a-chip. Additionally, efforts are being made globally to advance the utilization of organoid chips, such as the establishment of the European Organ-on-Chip Society in Europe.

A limitation of OoCs is their complex experimental setup [77], which can be avoided with clear guidelines or protocols. Cell medium changes also raise concerns about chip environments [77]. There is also the issue of using animal models to validate OoC systems initially [78]. To address this, OoC experts recommend forming well-established collaborations with developers, toxicologists, and pharmaceutical companies to explore alternative solutions.

### iPSCs(Induced Pluripotent stem Cells)

iPSCs are a recent development in the field of disease modeling. Having traits such as self-renewal and pluripotency, iPSCs can transform into various cells within the human body (Fig. 1); thus, reprogramming patient cells creates personalized medicine for specific diseases [79, 80]. The ability to produce a large batch of iPSCs with only a small number of patient samples is important [81, 82]. The objectives of iPSC models closely align with the 3R principle [83]. Replacing animal models in research while adhering to reduction and refinement principles is expected to be advantageous.

iPSCs are research to find cures for various diseases and are used as broad disease models (Table 2). For example, iPSCs from patients with Parkinson’s disease differentiate into midbrain dopaminergic neurons (DAns) in the substantia nigra pars compacta (SNpc), which can be used to model Parkinson’s disease on a cellular basis [43–45]. For cardiac diseases, which include a decrease in cardiomyocytes that leads to scar formation and ultimately heart function failure, there are existing studies that explore iPSCs for novel therapeutic cures [84]. iPSC-derived progenitors such as human HCN4 + and human ESC derived ROR2+, CD13+, KDR+, PDGFRα + cells later generate cardiomyocytes [47]. For cancer modeling using iPSCs, reprogrammed tumor specimens or iPSCs with premalignant or early genetic lesions can show the stages of cancer [49]. iPSCs from patients that are healthy and those with Alzheimer’s disease differentiate into the main brain cells, modeling the human brain with a functional blood barrier. Further research could drive drug discovery [9]. Studies of organ failure or dysfunction have shown that human iPSCs are useful. Research on lung regeneration has shown that endogenous and exogenous stem cells mediate therapeutic results [50]. Another study focused on the use of liver hepatoblasts, which could help alleviate hepatotoxicity through liver development and hepatic differentiation [85].

However, iPSCs are still in a relatively early developmental phase and have several limitations. Concerns for researchers regarding iPSCs is in vitro culture adaptation and tumorigenicity, the inability to completely reflect in vivo 3D environments, and the variation of differentiated cells depending on the protocol [86, 87]. Quality control of differentiated cells and influencing factors are crucial for iPSC researchers, impacting their applicability as medical models or treatments.

**Fig. 2:**
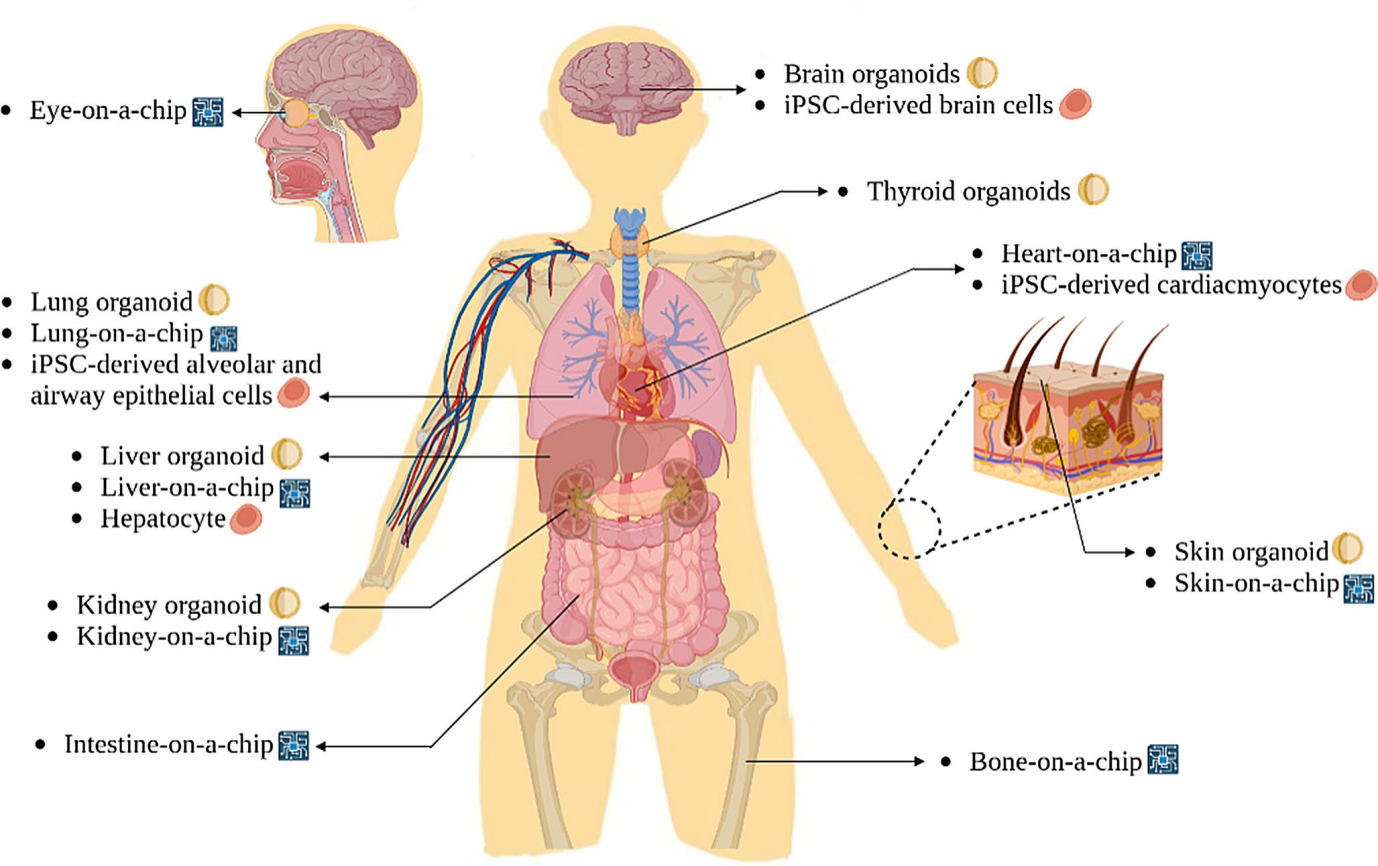
A BioRender diagram depicts diverse stem cell technologies for human organs

Figure 2 Human diagram showing multiple stem cell-related technologies that can be applied to various human organs.

## Limitations

Stem cell-related methodologies, such as organoids, are a very new technology in the field of animal alternative testing. In the early developmental stage, alternative stem cell models and technologies still require a few years of testing. Animal testing is still used today, owing to its historical role in safety and efficacy assessment. New alternatives have been presented; however, the uncertainty of these methods have caused most researchers to adhere to old protocols. In cases of complex diseases arising from various factors such as cardiovascular, neurodegenerative, and infertility, complete replacement by animal alternative testing methods may still be impractical. In such instances, it is crucial to concurrently employ animal experimentation alongside alternative testing methods utilizing organoids or stem cells to bolster data reliability. As a component of these endeavors, numerous researchers have undertaken disease modeling, such as stroke, utilizing brain organoids and cardiac organoids in in vitro experiments. The solution involves focusing on alternative testing methods [88]. By transforming old methods and creating alternatives, this shift could be the norm. There has already been a move toward that goal, as the FDA has established a cross-agency working group (The Alternative Methods Working Group) to promote various alternative methods, such as in vivo, in vitro, *in silico*, or system toxicology modeling [89]. In the 2021, FDA report titled “Advancing Regulatory Science at FDA,” the most prioritized area is identified as “Advancing Novel Technologies to Improve Predictivity of Non-clinical Studies and Replace, Reduce, and Refine Reliance on Animal Testing.”

## Conclusion

Given ongoing research in alternative stem cell-related methods, this appears promising to replace animal testing. These alternatives offer advantages for scientists and the public. However, it is important to acknowledge that iPSCs, organoids, and OoCs each have distinct strengths and limitations. With continued advancements and studies to further understand these issues, these limitations can be avoided.

## Data Availability

All data pertaining to this manuscript are included within the article.

## Abbreviations

FDA: Food and Drug Administration
OoC: organ-on-chip
iPSC: induced pluripotent stem cell
PSC: pluripotent stem cell
ESC: Embryonic stem cell
CDER: Center for Drug Evaluation and Research, GLP, Good Laboratory Practice
DAns: Dopaminergic neurons
SNpc: Substantia Nigra pars compacta
